# Whole Genome Resequencing Revealed the Effect of Helicase *yqhH* Gene on Regulating *Bacillus thuringiensis* LLP29 against Ultraviolet Radiation Stress

**DOI:** 10.3390/ijms24065810

**Published:** 2023-03-18

**Authors:** Weibo Ma, Xiong Guan, Ying Miao, Lingling Zhang

**Affiliations:** 1 State Key Laboratory of Ecological Pest Control for Fujian and Taiwan Crops, Key Laboratory of Biopesticide and Chemical Biology of Ministry of Education & Ministerial and Provincial Joint Innovation Centre for Safety Production of Cross-Strait Crops, College of Life Science, Fujian Agriculture and Forestry University, Fuzhou 350002, China; 2 College of Plant Protection, Fujian Agriculture and Forestry University, Fuzhou 350002, China; 3 College of Life Science, Fujian Agriculture and Forestry University, Fuzhou 350002, China

**Keywords:** *Bacillus thuringiensis*, whole genome resequencing, helicase, UV-resistance, gene knockout

## Abstract

*Bacillus thuringiensis* (Bt) is a widely used microbial pesticide. However, its duration of effectiveness is greatly shortened due to the irradiation of ultraviolet rays, which seriously hinders the application of Bt preparations. Therefore, it is of great importance to study the resistance mechanism of Bt to UV at the molecular level to improve the UV-resistance of Bt strains. In order to know the functional genes in the UV resistance, the genome of UV-induced mutant Bt LLP29-M19 was re-sequenced and compared with the original strain Bt LLP29. It was shown that there were 1318 SNPs, 31 InDels, and 206 SV between the mutant strain and the original strain Bt LLP29 after UV irradiation, which were then analyzed for gene annotation. Additionally, a mutated gene named *yqhH*, a member of helicase superfamily II, was detected as an important candidate. Then, *yqhH* was expressed and purified successfully. Through the result of the enzymatic activity *in vitro*, yqhH was found to have ATP hydrolase and helicase activities. In order to further verify its function, the *yqhH* gene was knocked out and complemented by homologous recombinant gene knockout technology. The survival rate of the knockout mutant strain Bt LLP29-Δ*yqhH* was significantly lower than that of the original strain Bt LLP29 and the back-complemented strain Bt LLP29-Δ*yqhH*-R after treated with UV. Meanwhile, the total helicase activity was not significantly different on whether Bt carried *yqhH* or not. All of these greatly enrich important molecular mechanisms of Bt when it is in UV stress.

## 1. Introduction

*Bacillus thuringiensis* (Bt), a gram-positive bacterium, is commonly used in microbial pesticides with the advantages of safety and good insecticidal effect [1]. As a popular biopesticide, Bt produces insecticidal crystal proteins (ICPs) that are effective against a variety of insect species, including Lepidoptera, Hemiptera, and Diptera [2,3,4]. However, the structure of insecticidal crystal proteins and the mechanism of action with pests need to be studied in depth [5]. As a biogenic insecticide, it has significant advantages over chemical insecticides [6]. External factors such as ultraviolet radiation can damage or degrade Bt strains and their toxin proteins, reducing the duration of Bt preparations and consequently limiting their effectiveness [3,7]. Therefore, developing transgenic Bt crops, screening of strains with strong resistance to UV, or improving melanin production are important for the application of Bt [8,9,10].

Whole genome resequencing is widely used in the research of plants and microorganisms [11,12]. Whole genome data have been reported for a wide range of Bt strains, providing a large amount of information for the research of Bt [13,14]. However, bioinformatics analysis of Bt in stress environments, especially UV stress environments, is still scarce. Therefore, the re-sequencing of highly UV stress-resistant strains and the discovery of variation information in gene sequences by sequence comparison can provide an effective method for screening UV resistance genes.

Ultraviolet (UV) is an invisible spectrum spanning wavelengths from 200 to 400 nm. UV radiation directly affects the survival of microorganisms. Therefore, it is essential to investigate the UV resistance mechanism of microorganisms. In *Bacillus*, melanin can attenuate the effect of UV exposure on Bt insecticide products and has been used as a protective agent [9]. Currently, several strains have been screened for the production of high levels of melanin, which can effectively increase the UV resistance of the strain [15,16,17]. In addition, algae and cyanobacteria have evolved various avoidance and repair mechanisms to protect themselves against the damaging effects of UV radiation to acclimate to enhanced UV-B radiation. For example, cyanobacteria can provide effective resistance to UV stress through secondary metabolites [18,19].

Ultraviolet radiation results in extensive DNA damage, with double-strand breaks (DSBs) as the most prominent formed of damage. Studies have shown that helicase have an important function in the repair of DNA damage. Helicase, a key macromolecular substance in the process of biomolecular metabolism, exists widely in various organisms. Helicase can convert double-stranded nucleic acids or structured nucleic acid strands to single-stranded nucleic acid, thereby mediating the metabolic activities of DNA or RNA [20,21]. It had been found that helicase had various biological functions. In cells, they were involved in DNA replication, repair, transcriptional recombination, RNA splicing, protein translation, genome stability, and other important molecular activities process [22,23,24]. According to its sequences, as well as structural and mechanistic features, helicase can be classified into six families [25]. Among them, the two largest families are called superfamily I (SFI) and superfamily II (SFII). SFI and SFII are monomeric, and the other four families are hexameric [26]. After being reported as a new gene involved in recombination and repair, DNA helicase was found in many bacteria, such as *Escherichia coli*, *Pseudomonas*, *Neisseria meningitides*, and *B. subtilis* [27,28,29,30]. DNA helicase had been found to participate in the stress response of Bt. The DNA helicase *recG* in *B. thuringiensis* is associated with UV resistance, and *recG* knockout strains are more sensitive to UV light [31]. A variety of RNA helicases have been identified in *B. cereus*, which can participate in stress reactions under heat, oxidation, and pH stress [32,33]. Whether other types of helicases also regulate the stress response of Bt remains to be determined.

In our previous study, UV-resistant Bt LLP29-M19 was obtained after the moquitocidal Bt strain LLP29 was exposed to UV for 19 generations. Bt LLP29-M19 showed re-sistance to UV radiation for up to 60 min [8]. In this study, the UV mutant strain Bt LLP29-M19 with high UV resistance was re-sequenced and compared with its original strain Bt LLP29. We found a helicase superfamily II member, *yqhH*, mutated in Bt LLP29-M19. To determine the function of *yqhH* in Bt UV resistance, the in vitro activity of yqhH was measured, and the strains of Bt LLP29 with and without *yqhH* were compared for UV resistance, as well as their DNA unwinding activities in different UV-B treatments. The present results could not only enrich the genomic data of Bt but also enhance the understanding of the UV resistance mechanism of Bt and provide an important theoretical basis for the field application of Bt.

## 2. Results

### 2.1. Sequencing Quality Control and Assembly of Bt LLP29-M19

Genomic DNA was extracted from the samples, and eligible libraries were sequenced by Illumina Hiseq 2500. A total of 7,302,812 raw reads were measured for the Bt LLP29-M19 strain. After quality control, 7,301,420 clean reads were obtained, and the GC content of the measured data was 35.61%. Using BWA software (version 0.7.10-r789), the obtained clean reads were matched to the Bt LLP29 reference genome; 98.45% of the reads were mapped to the reference genome; and 98.04% of the reads were properly mapped (Table S1). By analyzing the length of the inserted fragment, its distribution conforms to the normal distribution, indicating that the construction of the sequencing data library was not abnormal (Figure S1).

### 2.2. Differential Analysis of Resequencing Results

The Bt LLP29-M19 genome and LLP29 sequencing data were analyzed and compared in this study. Compared with the existing reference genome, the genome-wide heterozygosity ratio of Bt LLP29-M19 strain was 0.17%. Compared with the reference genome, the total number of SNP site variations was 1318; the sum of InDel mutations was 31; and the total number of SV-annotated variants was 206 (Table 1 and Table S2). For further analysis, the mutated genes were annotated into the database, resulting in 149 mutated genes. In addition, it was found that in most of the variant types, the variation mainly existed in the exonic region.

### 2.3. Functional Annotation of Variant Genes at the DNA Level

**Figure 1:**
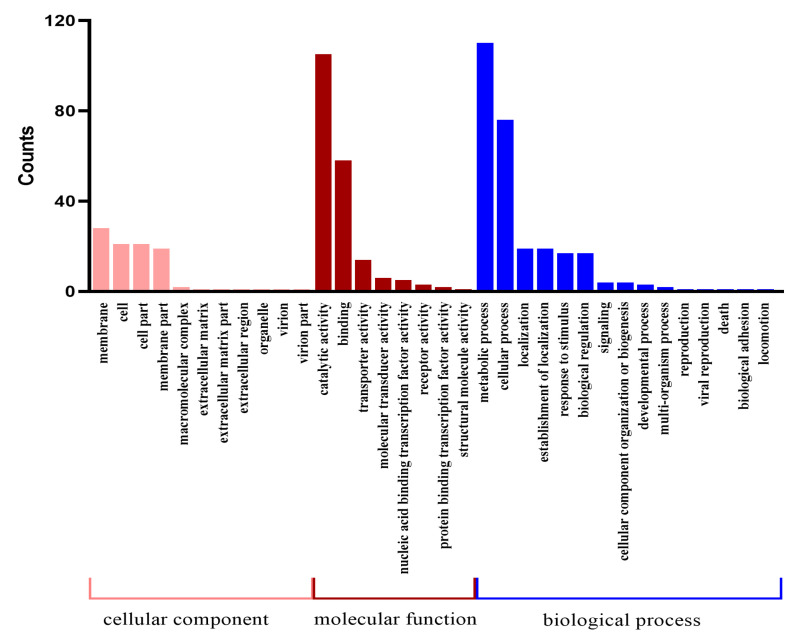
Variation gene GO annotation clustering.

**Figure 2:**
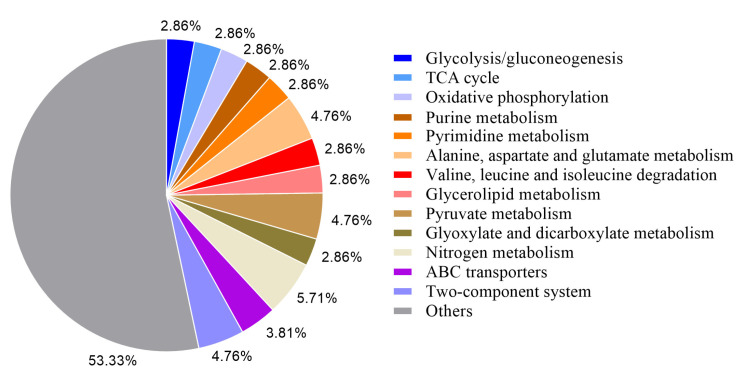
Classification of gene variations compared with KEGG database by blast.

Variations in the CDS region may cause changes in gene function, and the variant genes in Bt LLP29-M19 were annotated. A total of 567 variant genes were annotated by COG, among which the functional classes of genes with more variants were metabolic process (110), catalytic activity (105), cellular process (76) and binding (58) (Figure 1). At the same time, the obtained variant genes were subjected to KEGG analysis, and a total of 105 were annotated into different signaling pathways. The main annotated metabolic pathways of these variant genes were ABC transporters (173), two-component system (88), purine metabolism (70), and pyrimidine metabolism (64) (Figure 2).

### 2.4. UV-Related Gene Screening

Environmental changes can cause extensive DNA damage, and double-strand breaks (DSBs) are the most critical form of biological damage. In bacteria, homologous recombination repair is the most important repair method for DNA double-strand breaks. Studies have identified a number of helicase capable of participating in nucleotide excision repair, such as *pcrA* and *recQ* [34,35]. By GO and KEGG analysis, a total of 149 genes were annotated in the variant genes. We focused on the helicase genes. We identified four helicase genes mutated in Bt LLP29-M19 by sequence comparison. We found that one of the genes had a base mutation from C to T at the 265th position, and the amino acid changed from glutamine to terminator (Figure S2). By gene annotation, it was named *yqhH*, which belongs to the SNF2 family of DNA decapping enzymes. Therefore, to investigate the function of the helicase in Bt anti-UV, we selected the *yqhH* gene for in-depth analysis.

### 2.5. Cloning and Expression of yqhH Gene

**Figure 3:**
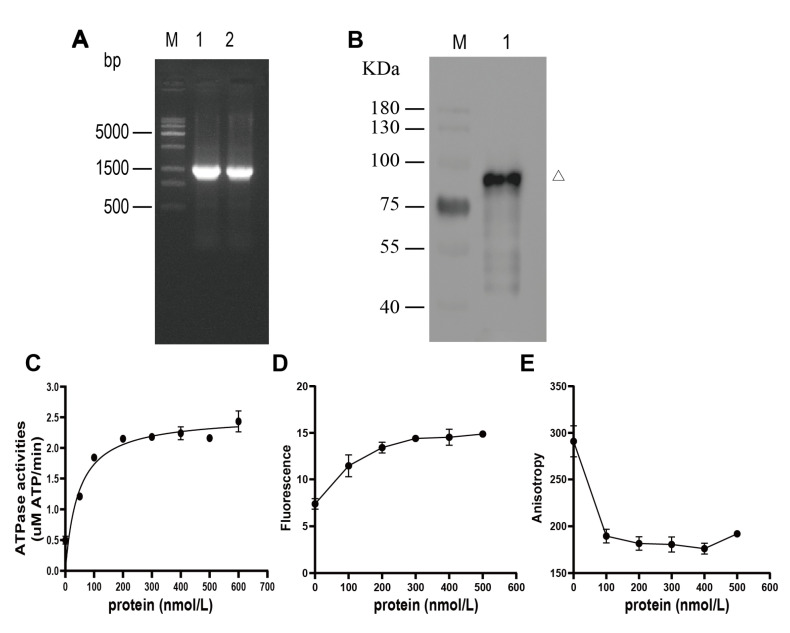
Cloning, protein expression and activity analysis of *yqhH* gene. (**A**) PCR product of fragment *yqhH*. Lane M: 15K DNA marker; lane 1, 2: *yqhH* PCR product. (**B**) Western blot analysis of yqhH helicase. Lane M: Protein mark; lane 1: Purified yqhH. (**C**) ATP hydrolysis activity assay of yqhH helicase. (**D**) Changes in the fluorescence intensity of the substrate donor in the presence of ATP. (**E**) Fluorescence resonance energy transfer changes in yqhH unwound substrates in the presence of ATP. In (**C**–**E**), data are the mean values of three technical replicates ±SD.

To analyze the function of the *yqhH* gene in vitro, a *yqhH* gene of size 1.5 Kb was first obtained by PCR (Figure 3A). Then, the expressed yqhH was purified by protein Ni-NTA resin kit after induction by 0.5 mM IPTG. To test the accuracy of the purified protein, the results were analyzed by Western blot, which showed that the molecular weight of the yqhH fusion protein was about 80 kDa, as expected (Figure 3B).

### 2.6. ATP Hydrolysis Activity of yqhH Helicase

To elucidate the function of *yqhH*, an ATP hydrolysis activity assay was first performed. In presence of ATP, yqhH protein could catalyze the hydrolysis of ATP, which indicated that yqhH helicase was a DNA-dependent ATPase. Meanwhile, the ATP hydrolysis activity increased in a gradient with the increase in enzyme concentration. When the helicase concentration reached a certain amount, the ATP hydrolysis activity reached saturation (Figure 3C). These results indicate that yqhH expressed in vitro can hydrolyze ATP and have ATP hydrolysis activity.

### 2.7. Unwinding Activity of yqhH Helicase

In the helicase kinetics assay, different concentrations of yqhH helicase were added into the unwinding reaction, respectively. As the concentration of yqhH increased, the fluorescence intensity of the substrate donor increased while the relative intensity of fluorescence polarization decreased (Figure 3D,E). In addition, the helicase activity increased with the increase in yqhH concentration. When the enzyme concentration reached a certain amount (400 nmol), the helicase activity reached a maximum, indicating that the helicase activity was concentration-dependent, which suggested that yqhH expressed in vitro had helicase activity.

### 2.8. Knocking out yqhH in Bt LLP29

**Figure 4:**
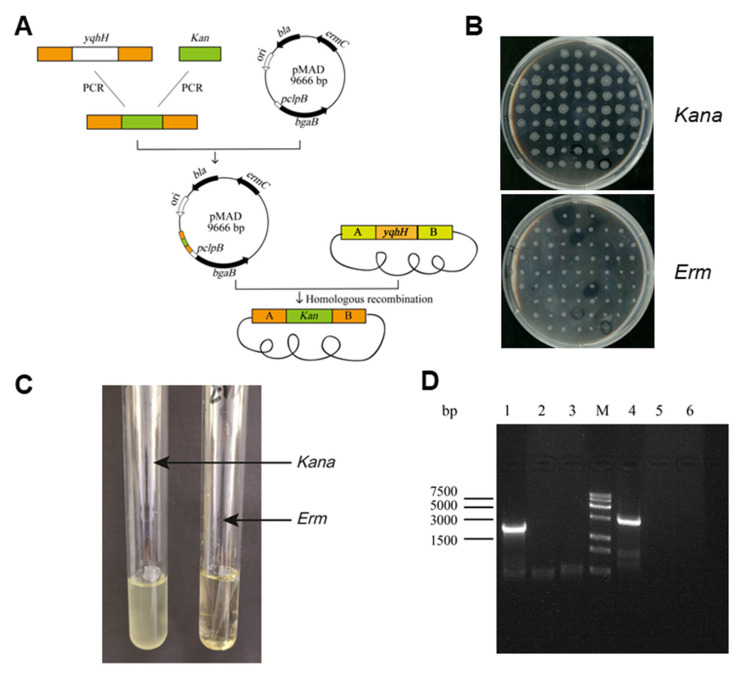
Construction of knockout strains. (**A**) Flow chart of Bt LLP29-△*yqhH* defective strain. (**B**) *Kana* and *Erm* plates screen for defective strains. (**C**) *Kana* and *Erm* liquid LB verification defective strains. (**D**) PCR cross-validation of Bt LLP29-Δ*yqhH*; lane M: 15 K DNA marker; The templates for 1–3 are Bt LLP29-Δ*yqhH*,Bt LLP29,H_2_O, and the primers are F1/R1; 4–6 templates are in the same order as 1–3 templates, and the primers are F2/R2.

To knock out the *yqhH* gene of Bt LLP29, experiments were performed by homologous recombination technique (Figure 4A). The recombinant plasmid was transformed into Bt LLP29 competent cells by electroporation, and the strain with *Kana* resistance but no *Erm* resistance was reconstituted into by high temperature (Figure 4B). The knockout strain was further characterized by expanded culture, and the Bt LLP29-Δ*yqhH* strain could be grown normally in a Kana antibiotic-containing medium (Figure 4C). To confirm the successful knockout of *yqhH*, total DNA of the mutant strain was extracted and examined by PCR using specific primers (Figure 4D). It was also indicated that *yqhH* was successfully knocked out in strain Bt LLP29-Δ*yqhH* by sequence alignment.

### 2.9. Complementation of yqhH in Bt LLP29-ΔyqhH

**Figure 5:**
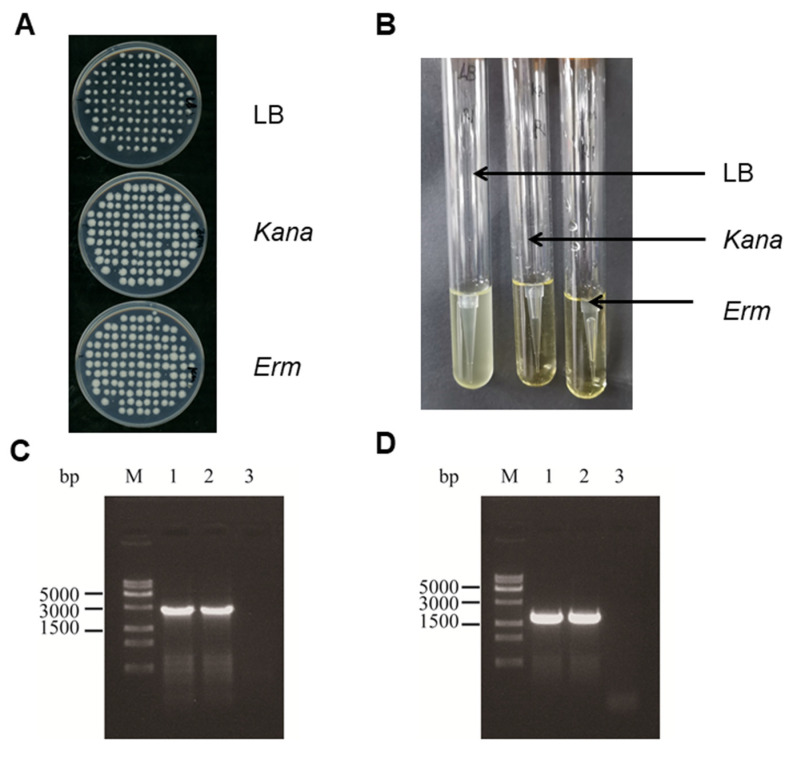
Screening and validation of Bt LLP29-△*yqhH*-R strain. (**A**) *Kana* and *Erm* plates screen for defective strains. (**B**) *Kana* and *Erm* liquid LB verification defective strains. (**C**) PCR cross-validation of Bt LLP29-Δ*yqhH*-R; lane M: 15K DNA marker; The templates for 1–3 are Bt LLP29-Δ*yqhH*-R,Bt LLP29,H_2_O, and the primers are F1*/R1*. (**D**) PCR cross-validation of Bt LLP29-Δ*yqhH*-R; lane M: 15K DNA marker; The templates for 1–3 are Bt LLP29-Δ*yqhH*-R,Bt LLP29,H_2_O, and the primers are F2*/R2*.

To further investigate the function of *yqhH*, the gene was also restored in this study. The recombinant plasmid was desalinized and electroporated into Bt LLP29-Δ*yqhH* competent cells. After high-temperature recombination screening, a new strain was obtained that was resistant to neither *Erm* nor *Kana* (Figure 5A). The complementation strain was then further characterized by expanded culture, and the Bt LLP29-Δ*yqhH*-R strain could only grow normally in antibiotic-free medium (Figure 5B). To confirm the successful complementation of target gene *yqhH* in Bt LLP29-Δ*yqhH*, the total DNA of selected strain was extracted, and the fragments of F1*R1*, and F2*R2* were amplified and detected (Figure 5C,D). After sequencing analysis, the amplified sequence had a homology of 99% with the previously reported sequence, indicating that the complementary strain Bt LLP29-Δ*yqhH*-R was successfully obtained.

### 2.10. Growth Curves and UV-B Treatment Phenotype

**Figure 6:**
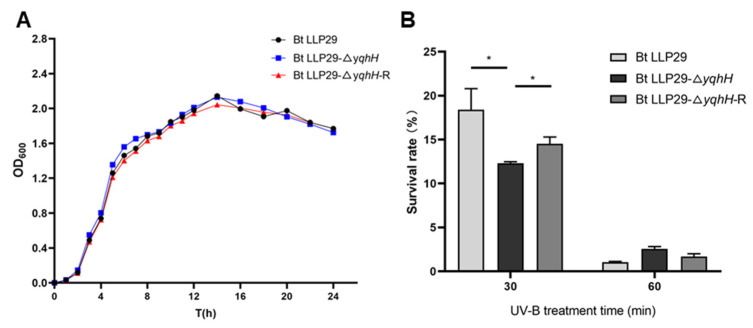
The growth curve and phenotypes of different Bt strains. (**A**) The growth curve. (**B**) The UV-B assay. Data are the mean values of three technical replicates ± SD. * *p* < 0.05 (*t*-test) indicates a significant difference between the original strain and mutants.

To detect the effect of *yqhH* on the growth of Bt, the growth curves of three strains were first analyzed. The results showed that similar growth curves were detected in the different tested Bt strains with minimal difference. At the early growth stage, Bt LLP29-Δ*yqhH* grew slightly faster than Bt LLP29 and Bt LLP29-Δ*yqhH*-R. However, three tested strains entered a stationary phase with an OD_600_ of about 2.0 after 14 h (Figure 6A).

To test the effect of *yqhH* on Bt in UV, the following experiments were performed. Three strains, Bt LLP29, Bt LLP29-Δ*yqhH*, and Bt LLP29-Δ*yqhH*-R, were treated with UV irradiation to observe the survival rate of the strains for 0 min, 30 min, and 60 min, respectively. The survival rates of three tested strains decreased with the increase in UV treatment time. However, the survival rate of Bt LLP29-Δ*yqhH* was significantly lower than both of Bt LLP29 and Bt LLP29-Δ*yqhH*-R when UV treatment was performed for 30 min. After UV treatment for 60 min, the survival rate of three strains were all lower than 5%, but the survival rate of Bt LLP29-Δ*yqhH* was slightly higher. The results indicate that the *yqhH* gene can regulate the ability of Bt strains to resistance to UV (Figure 6B).

### 2.11. Unwinding Activity In Vivo

**Figure 7:**
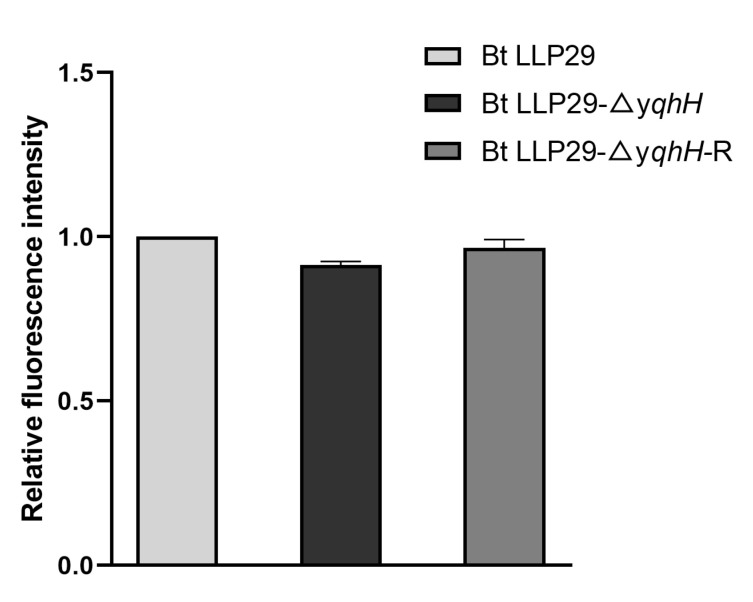
Unwinding activity of different strains. Data are the mean values of three technical replicates ± SD.

To detect the helicase activity of yqhH, an unwinding activity comparison was carried out in the tested Bt strains. However, no significant differences in the unwinding ability were found between the knockout and wild-type strains and the complementary strains carrying *yqhH*. After deletion of *yqhH*, the helicase activity of Bt LLP29 was similar to that of Bt LLP29 and its complementary strain Bt LLP29-Δ*yqhH*-R (*p* > 0.05). The helicase activity of Bt LLP29-ΔyqhH-R was also not found to be significantly different when *yqhH* was complemented (Figure 7). These results suggest that *yqhH* is not significantly involved in the helicase activity of Bt LLP29. However, there may be other factors besides *yqhH* that have helicase activity.

## 3. Discussion

*Bacillus thuringiensis* is a widely used microbial insecticide. The adverse external environment, such as ultraviolet radiation, can destroy or degrade Bt strains and their toxin proteins, which can shorten the shelf life of Bt products and greatly limit their application. How to effectively select the critical genes for UV resistance will provide invaluable information for understanding the molecular mechanism of UV stress resistance in Bt [15,36]. With the development of sequencing technology, genome resequencing has offered a convenient way to identify mutated genes between resistant strains and original strains [37].

A large number of differential genes can be identified by gene sequencing, which provides an effective method for identifying critical genes that regulate stress accordingly [38,39]. In this study, the Illumina Hiseq 2500 was used to re-sequence the genome of UV-resistant strain LLP29-M19, and 149 mutated genes were found. These genes were functionally annotated and were mainly associated with genes for glucose conversion and synthesis, signal transduction, nucleotide excision repair, and cellular components. These results suggest that UV light affects the growth of Bt through the synthesis of glucose in Bt and the DNA replication pathway.

UV has been shown to cause direct damage to DNA molecules [40]. Previous studies have found that unwinding enzymes play an important role in DNA damage repair. Helicase is present in most organisms and plays various functions such as DNA replication, repair, recombination, and transcription [23,41]. Many of the helicases in *B. subtilis* and *E. coli* have been studied intensively [42,43]. In *B. subtilis*, it had been proved that *pcrA, recQ*, and *recG* helicases had ATPase and DNA unwinding activities and played an essential role in maintaining the stability of genes [29,35,44]. In this study, we found three helicase genes that were mutated after UV treatment, suggesting that these genes may be involved in the UV response. Additionally, the *yqhH* gene was selected for functional analysis. The enzymatic activity of yqhH in vitro was assayed by FRET, and it was in agreement with the gene annotation results that yqhH was able to hydrolyze ATP and unwind double-stranded DNA (Figure 3C–E). However, the specific mechanism of yqhH protein deconvolution needed to be further investigated. We further knocked down the *yqhH* gene in Bt and examined the changes in total protein activity in the different tested strains, while no significant changes were observed in the unwinding enzyme activity (Figure 7). Therefore, we speculate that other similar unwinding enzymes exist in vivo that could complement the function of *yqhH* knockdown.

The helicase regulates the microbial response to stress, and the same family helicases can perform different functions [31,32]. In the UV treatment test, the survival rate of tested Bt strains decreased after UV stress. UV was found to be able to significantly reduce the survival rate of Bt strains, but there were variations between different Bt strains (Figure 6B). Consistent with previous results, UV could affect the growth of microorganisms [17,36], which indicated that yqhH helicase could play a role in the repair of cells after damage. However, the knockout mutant was more sensitive to UV and grew more slowly than the other Bt strains harboring *yqhH* (Figure 6B). This result indicates that the cells are subjected to UV irradiation, and the deletion of *yqhH* affected the normal unwinding of DNA, stalling replication and affecting cell growth. Additionally, the survival of the knockout mutant was obviously reduced when it was treated with UV for 30 min, indicating that the unwinding enzyme might be involved in the response to stress at the early stage of stress.

In addition, there are some genes among the mutated genes that might affect the ability of Bt to function in the UV environment. We found that the gene encoding the insecticidal crystal protein cry4B also had an SV mutation, suggesting that UV may affect the insecticidal ability of Bt, and the exact mechanism needs to be investigated. In addition, a regulatory protein, *ylbF*, had an SV mutation. Studies in *Bacillus subtilis*, suggest that *ylbF* is required for both the sensory state and spore formation. *ylbF* deletion leads to a significant reduction in the expression of *comK*, a key regulator of the sensory state [45]. In strain Bt LLP29-M19, the tyrosine recombinase *xerC* gene was also mutated in SV. In most bacteria, *xerC* and *xerD* act on the replication endpoint region of the chromosome to dissociate dimers into monomers in a homologous recombination manner [46,47]. Some antioxidant genes were also mutated among the mutant genes, such as *gltA* and *glsA*. In conclusion, all of these results could not only enrich the genomic information of Bt but also provide the basis for further study of the regulatory mechanism of UV resistance.

## 4. Materials and Methods

### 4.1. Tested Strains

Bt LLP29 was isolated from Magnolia denudate in the laboratory [48]. Bt LLP29-M19 is a high UV-resistant Bt strain with stable genetic characteristics [8]. *E. coli* PMAD and *E. coli* PSTK were kindly provided by the Institute of Plant Protection, Chinese Academy of Agricultural Sciences.

### 4.2. Sequencing, Assembly and Analysis of Genome Sequences

Genomic DNA of acceptable quality was fragmented by ultrasonic broken method. The fragmented DNA was then subjected to fragment purification, end-repair, 3′- end addition of A, and ligation of sequencing connectors, followed by agarose gel electrophoresis for fragment size selection and PCR amplification to form sequencing libraries. The quality-checked libraries were sequenced by Illumina HiSeq 2500. Reads with adapters are removed; low quality values are filtered to ensure data quality. The qualified reads were analyzed for mapping by Burrows-Wheeler Aligner (BWA) software (version 0.7.10-r789) with Bt LLP29 genome as reference [49]. SNP and small InDel detection and annotation were conducted using GATK software (version 3.4) and SnpEff software (version 4.3), respectively [50,51]. The BLAST variant genes were compared with functional databases such as GO and KEGG to analyze the functions of these genes [52,53].

### 4.3. Cloning, Expression, and Purification of yqhH

To generate the *yqhH* fragment for cloning, the DNA was amplified using the Bt LLP29 genome. The target fragment was cloned by a TA cloning kit to obtain the positive strain, and the plasmid was extracted, identified by PCR and enzyme digestion, and the correctly sequenced plasmid was named pMD19T-*yqhH*. Additionally, the pMD19T-*yqhH* recombinant plasmid was digested with BamHI/EcoRI and then ligated into the Pet-32a expression vector. Sequenced clones were transformed into *E. coli* BL21 cell to express recombinant yqhH. After induced by 0.5 mM IPTG, the target protein was purified using a Protein Ni-NTA Resin reagent kit (TransGen Biotech, Beijing, China). Finally, the purified yqhH protein was detected by SDS-PAGE, and the protein concentration was determined by BCA method.

### 4.4. ATPase Activity Assay

ATPase activity was measured using the ATPase assay kit (Innova Biosciences, Cambridge, Britain): 100 μg of the extracted protein solution was added to 100 μL of substrate buffer (0.1 M Tris-HCl, 5 mM MgCl_2_, 1 mM ATP, 4 nM dsDNA). The reaction was carried out at 37 °C for 20 min, and the Goldmix was added to terminate the ATP hydrolysis reaction. Then, the stabilizer was added after 2 min. After 30 min at room temperature, the absorption value was measured at 635 nm wavelength. The enzymatic activity (μM/min) of the helicase was defined as the amount of enzyme required to catalyze the hydrolysis of 1 mol of substrate per minute.

### 4.5. Unwinding Activity Assay

The DNA-melting activity of the enzyme was analyzed by Fluorescence Resonance Energy Transfer (FRET) [54]. Experiments were performed referring to previously described methods with modifications [55]. The reaction buffer (25 mmol/L Tris-HCl, 1 mmol/L MgCl_2_, 50 mmol/L NaCl, 0.1 mM DTT, 1 mmol/L ATP, pH 7.5) with 2 nmol/L dsDNA (5′H-A/3′F-B) was added into different concentrations of yqhH enzyme (total 200 μL), and then the reaction was conducted at room temperature for 20 min; finally, the fluorescence of the sample was detected at a 525 nm wavelength by a microplate reader signals. All quantitative results were obtained from triplicate assays and analyzed using GraphPad Prism 8.

### 4.6. Construction of the Gene Knockout Vector

To construct the *yqhH* gene knockout vector, standard cloning procedures were used. *E. coli* pMAD was selected as the knockout vector, and the Kana resistance gene was used to replace the target gene to construct a *yqhH* gene knockout strain. According to the Bt LLP29 genome, specific primers *yqhH*-A1, *yqhH*-A2, *yqhH*-B1, and *yqhH*-B2 were designed. Primers *yqhH*-K1 and *yqhH*-K2 were designed for kanamycin sequences in *E. coli* PSTK (Table S3). Using Bt LLP29 total DNA and *E. coli* PSTK total DNA as templates, the specific primers *yqhH*-A1/A2 and *yqhH*-B1/B2 were used to amplify the fragment A and B of *yqhH*, and primer *yqhH*-K1/K2 was employed to amplify the resistant fragment K. After purification, the PCR products were ligated by ClonExpress II One Step Cloning Kit (Vazyme, Nanjing, China), and the gene knockout vector PMAD-Δ*yqhH*-AKB was successfully constructed. The positive cloned plasmids were confirmed by enzyme digestion and sequencing and then transferred to *E. coli* Trans110 competent cells for demethylation, and the plasmid PMAD-Δ*yqhH*-AKB-Trans110 was extracted for verification and desalinization.

### 4.7. Preparation and Transformation of Bt LLP29 Competent Cells

Preparation and transformation of competent Bacillus bacteria were performed as previously described [56]. The activated Bt LLP29 strain was transferred to a 200 mL LB medium and then incubated at 30 °C until the value of OD_600_ was from 0.5 to 0.7. Cells were placed on ice for 30 min and collected by centrifugation at 4 °C and 7000 rpm for 10 min. After 4 washes with SG (0.5 m/L Mannitol + 10% Glycerol), the pellet was gently pipetted with 200 uL SG and then transferred to a 1.5 mL EP tube. Taking 20 μL plasmid to 100 μL competent cells, the mixture was placed on ice for 10 min before electroporation. Transfection was performed by electroporation using a MicroPulser Electroporator (Bio-Rad, Shanghai, china). After successfully shocked, the pellet was added to 1800 μL of LB medium. After incubated at 30 °C and 150 rpm for 3 h, it was centrifuged at 12,000 rpm for 3 min; the supernatant was discarded; and then the cells was spread on an LB solid medium (*Kana* + *Erm*), following culturing at 30 °C for 24 h.

### 4.8. Homologous Recombination and Identification

Knockout gene homologous recombination and identification of knockout strains were performed as previously described [31]. The mutant strain was obtained by high temperature homologous recombination reaction. Positive clones were activated and then transferred to an LB medium without antibiotics at 43 °C. When the value of OD_600_ was 0.6, the cells were again transferred to a fresh LB medium without antibiotics, and this was repeated 4 times. The bacterial solution was spread on a *Kan* plate and incubated at 30 °C for 12 h. Individual clones were then selected and loaded onto Kan and *Erm* plates for screening. To verify the mutant strain, *yqhH*-F1, *yqhH*-R1, *yqhH*-F2, and *yqhH*-R2 primers were used to amplify the fragments of *yqhH*-F1/R1, *yqhH*-F2/R2 (Table S3). Total DNA of Bt LLP29 was used as a negative control. PCR products were sequenced and analyzed (Sangon Biotech, Shanghai, China).

### 4.9. Construction and Verification of yqhH Deletion Complementary Strain

The deletion-complementing strain Bt LLP29-Δ*yqhH*-R was constructed according to the method of gene knockout. Using Bt LLP29 total DNA as a template and *yqhH*-A1/*yqhH*-B2 as primers, the target sequence *yqhH*-AB was amplified and then connected to construct a recombinant vector PMAD-*yqhH*-AB-R. The verified correct plasmid was demethylated and desalinated, and then the plasmid was electroporated into the competent cells of the defective Bt LLP29-Δ*yqhH*. The positive transformants were screened for complementary strains Bt LLP29-Δ*yqhH*-R after high temperature recombination at 43 °C. For further verification, the Bt LLP29-Δ*yqhH*-R total DNA was used as the template, and the *yqhH*-R-F1*, *yqhH*-R-R1* and *yqhH*-R-F2*, *yqhH*-R-R2* primers were used to amplify the fragment F1*/R1*and F2*/R2*, respectively. The PCR product was verified by sequencing analysis.

### 4.10. Growth Curve Assay

To detect the effect of the knockdown of *yqhH* on the growth of Bt, the growth curves of Bt LLP29, Bt LLP29-Δ*yqhH*, and Bt LLP29-Δ*yqhH*-R were measured. The tested strains were grown in 100 mL LB media at 30 °C to an OD_600_ of 1.0 and then transferred to 250 mL LB media at 1% and cultured in a shaker at 30 °C. The OD_600_ was measured every 1 h from 1 to 12 h and every 2 h from 12 to 24 h. Finally, the growth curve was plotted, with the growth time as the abscissa and the OD_600_ as the ordinate.

### 4.11. UV-B Treatment Experiment

The Bt LLP29, Bt LLP29-Δ*yqhH*, and Bt LLP29-Δ*yqhH*-R strains were activated and transferred to 100 mL liquid LB for incubation until the stable phase. According to the previous method [31], 2 mL of each strain was placed in a 6-well plate and treated with UV-B in a Scientz03-II UV Crosslinker for 0, 20, and 40 min. Samples were removed, and the plates were coated with a 10*^−^*^4^ dilution of the bacterial solution. Three parallel replicates were set up, and the experiment was repeated three times. The survival rate of the three strains treated with UV at different stress times was calculated by CFU counting.

## Figures and Tables

**Table 1:** Comparative analysis of genome variation in the Bt LLP29-M19 strain.

Variation Type	Number of Variants	Sum
SNP annotation		
Genome-wide heterozygous ratio (%)	0.177	
Total number of SNP loci	1318	
InDel annotation		
Sum of insertion mutations	14	31
Total deletion mutation	17	
SV annotation		
Deletion	35	206
Inversion	145	
inversion	26	

## Data Availability

Not applicable.

## Supplementary Materials

The following supporting information can be downloaded at: https://www.mdpi.com/article/10.3390/ijms24065810/s1.
