# Supplementary material for: Whole Genome Resequencing Revealed the Effect of Helicase yqhH Gene on Regulating Bacillus thuringiensis LLP29 against Ultraviolet Radiation Stress
Source: Int J Mol Sci. 2023 Mar 18;24(6):5810. doi: 10.3390/ijms24065810 (PMC10054049; doi:10.3390/ijms24065810)
Supplement: Supplementary file 1 [file ijms-24-05810-s001.zip › ijms-2213068-supplementary.pdf]

Supplemental Figures and Tables

Table S1. Comparison statistics with reference genome Bt LLP29.

Sample	Total-Reads	Mapped (%)	Properly-Mapped (%)
LLP29-M19	14,602,840	98.45	98.04

Total-Reads: The number of Clean Reads; Mapped (%): The percentage of Clean Reads mapped to the reference genome in all Clean Reads; Properly-mapped (%): The paired-end sequencing sequences are mapped to the reference genome and the distance conforms to the length distribution of sequencing fragments.

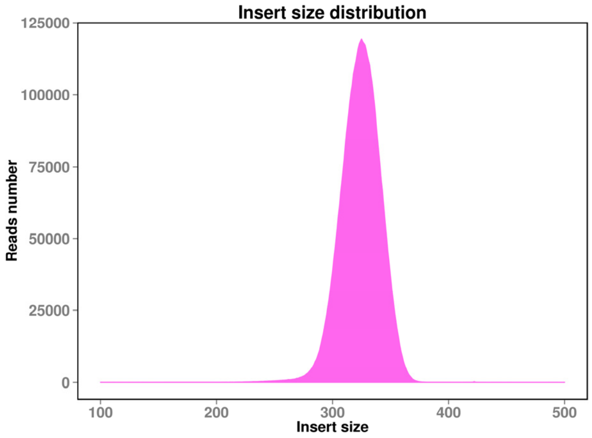

Figure S1. Distribution of Bt LLP29-M19 inserts. The abscissa is the length of the inserted fragment, and the ordinate is the number of corresponding reads.

Table S2. Statistics of SNPs detected by Bt LLP29-M29 and the reference genome.

SNP Number	Transition	Transversion	Ti/Tv	Heterozygosity	Homozygosity	Het-Ratio
1318	889	429	2.07	1085	233	0.17%

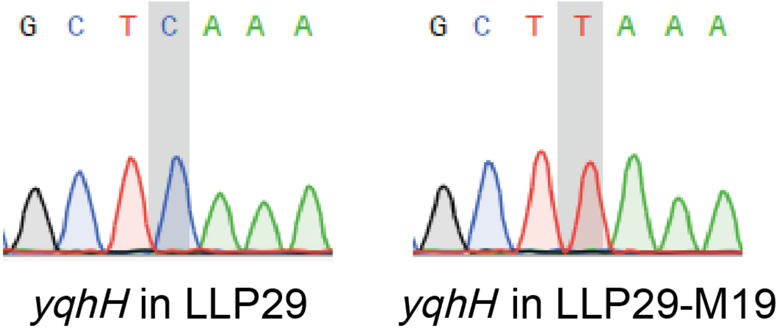

Figure S2. Sequence alignment of *yqhH* gene.

Table S3. Primers for vector construction, knockout and complementation, and validation of helicase activity.

yqhH -F	CGGGATCCATGAATGTCGATATTTCCGTAGATCG
yqhH -R	GGAATTCTCATGTTGCTGCATAGCCTTGC
5'H-A	H-GCACTGGCCGTCGTTTTACGGTCGTGACTGGGAAAACCCTGGCG
3'F-B	TTTTTTTTTTTTTTTTTTTTTTTTTCCAAGTAAAACGACGGCCAGTGC-F
yqhH -A1	CTAGAAGCTTCTGCAGACGCGTCGACGCTCCTTCTAAAGCAGAAGG
yqhH -A2	CACCTCAAATGGTTCGCTGCAAGAAATTTTCGCGCAATC

yqhH -B1	GAGCGCCTACGAGGAATTTCGATCTACGGAAATATCGAC
yqhH -B2	GCCTCGCGTCGGGCGATATCGGATCCCATGACTTGCTAACCATTCG
yqhH -K1	GATTGCGCGAAAATTTCTTGCAGCGAACCATTTGAGGTG
yqhH -K2	GTCGATATTTCCGTAGATCGAAATTCCTCGTAGGCGCTC
yqhH-F1	ATGTGAGCGAGTAAGAGTGG
yqhH-R1	GATGGAGTGAAAGAGCCT
yqhH-F2	TGAAACCCAGGACAATAA
yqhH-R2	CCTCTTCTGCTCCAACTTCA
yqhH-R-F1*	TGTGAGCGAGTAAGAGTGGC
yqhH-R-R1*	GAATGGTAAAGCGATTCTAGC
yqhH-R-F2*	GAACAGGAGTAGCGGTTAGT
yqhH-R-R2*	CGAAAGATTCCACACATTTCA

F: fluorescein, H: hexachlorofluorescein.
